# Enhancing essential amino acid bioavailability of soy protein through *Streptococcus thermophilus* ST4 supplementation

**DOI:** 10.3389/fmicb.2025.1740277

**Published:** 2026-01-06

**Authors:** David Agus Setiawan Wibisono, Chiou-Yeong Saw, Bo-Long Jian, Der-Kai Lau, Wei-Jen Chen, Hui-Fang Chu, Cheng-Yen Liu, Chi-Fai Chau

**Affiliations:** 1 Department of Food Science and Biotechnology, National Chung Hsing University, Taichung, Taiwan; 2 Research and Development Department, Syngen Biotech Co., Ltd., Tainan, Taiwan

**Keywords:** amino acids, high-protein diet, plant proteins, probiotics, *Streptococcus thermophilus*, validation

## Abstract

**Background:**

Soy protein is a popular plant-based protein source, but is nutritionally limited by its incomplete essential amino acid profile and lower bioavailability relative to animal proteins. Emerging evidence suggests that probiotics may improve the absorption of amino acids from plant proteins.

**Methods:**

Our research evaluated the ability of *Streptococcus thermophilus* ST4 to improve the soy protein nutritional quality by increasing the bioavailability of essential amino acids, using *in vitro* digestion models, as well as animal experiments.

**Results:**

After 4 h, supplementation with *S. thermophilus* ST4 increased amino acid concentrations *in vitro*. Total amino acids rose by up to 96.9%, essential amino acids by 69.1%, and non-essential amino acids by 124.4%. Afterward, rats fed either a standard or high-protein diet received *S. thermophilus* ST4 at doses of 1 × 10^7^ or 1 × 10^9^ CFU/day. High-dose treatment significantly elevated serum total amino acids by 29.7–32.4% and essential amino acids by 37.7–43.2% (*p* < 0.01). Postprandial analysis further confirmed a 62.0% increase in essential amino acids in the high-protein group following probiotic supplementation.

**Conclusion:**

*S. thermophilus* ST4 supplementation improved the nutritional quality of soy protein by enhancing essential amino acid bioavailability. These findings support its application as a probiotic for improving plant-based protein utilization, offering a practical dietary strategy to address amino acid limitations in vegetarian and high-protein diets.

## 1 Introduction

Plant-based proteins have earned widespread attention, propelled by improvements in processing technologies, increased availability, and growing awareness of their health-promoting properties. Numerous studies have linked sustained intake of plant proteins with favorable health effects (Lynch et al., 2018). Nevertheless, plant proteins generally fall short in terms of digestibility and essential amino acid completeness when compared to their animal-derived counterparts (Hoffman and Falvo, 2004). This discrepancy is reflected in their lower Digestible Indispensable Amino Acid Score, unlike animal proteins that typically exceed 100. The limitation of plant proteins is largely attributed to anti-nutritional factors. These factors impair enzymatic hydrolysis and hinder the intestinal absorption of amino acids (Herreman et al., 2020).

To overcome these nutritional limitations of plant proteins, strategies such as enzymatic hydrolysis, fermentation, and genetic modification have been investigated. Among these, probiotics have gained popularity as viable agents for enhancing amino acid profiles in plant-based proteins, largely due to their secretion of proteolytic enzymes. Additionally, probiotics produce other hydrolytic enzymes, such as xylanases, amylases, and glycosidases, that aid in breaking down protein-polyphenol complexes and facilitating macronutrient digestion (Yakubu et al., 2022). Previous research further supports this notion, showing that probiotic strains increased the concentration of free amino acids and α-amino nitrogen, thereby improving the digestibility of pea and soy proteins relative to controls (Marttinen et al., 2023). Most current studies still focus on general protein hydrolysis, with limited exploration of how probiotics affect the bioavailability of individual essential amino acids. The dose-dependent effects of probiotics on serum amino acid concentrations remain insufficiently explored.

The distinct physiological capabilities of lactic acid bacteria in protein hydrolysis are well recognized (Savijoki et al., 2006). *Streptococcus thermophilus*, a probiotic species commonly employed as a starter culture in dairy fermentation, particularly yogurt, has been recognized for its rapid acidification, established safety profile, and compatibility with co-cultures such as *Lactobacillus* spp. (Uriot et al., 2017). Beyond its traditional role, *S. thermophilus* has also shown independent probiotic potential, including enhancement of gut barrier integrity and support for intestinal colonization (Ma et al., 2022). Despite these benefits, its application as a standalone probiotic has been underexplored, particularly in non-dairy systems like plant protein fermentation. Preliminary studies suggest that *S. thermophilus* may aid proteolysis and enhance amino acid availability (Champagne et al., 2009). Yet direct evidence for its role in improving essential amino acid bioavailability from plant-based proteins is still limited and inconclusive.

Given these considerations, further investigation is warranted to clarify how *S. thermophilus* may enhance the nutritional benefits of plant-based proteins. This study evaluated its effects on amino acid composition, with a particular focus on its ability to promote protein hydrolysis and improve essential amino acid availability. The research determined whether this probiotic was capable of increasing serum essential amino acids using *in vitro* digestion assays and animal models. *S. thermophilus* ST4 was administered at varying doses across two distinct dietary regimens in the *in vivo* study, thereby offering practical insights into the probiotic’s role in promoting more balanced and nutritionally adequate diets. A postprandial analysis was also conducted to examine the immediate effects of *S. thermophilus* ST4 on amino acid absorption during active digestion.

## 2 Materials and methods

### 2.1 Samples

A purified powdered strain of *S. thermophilus* ST4 (1 × 10^11^ CFU/g) was used in this study. Strain identity was confirmed through molecular probing with 16S rRNA gene primers (F: 5′-AGAGTTTGATCCTGGCTCAG-3′; R: 5′-ACGGTTACCTTGTTACGACTT-3′), alongside morphological characterization (Supplementary Figures S1, S2; Supplementary Table S1). Soy protein isolate (ISP) powder, containing 92% protein (dry weight basis), was used as a plant protein substrate. Both the probiotic and ISP were sourced from Syngen Biotech Co., Ltd. and stored at −20 °C in airtight containers.

### 2.2 Probiotic doses

For *in vivo* administration, probiotic doses were selected based on common concentration ranges used to evaluate dose-dependent physiological effects and to reflect typical levels found in commercial products (10^6^–10^10^ CFU/day) relevant to functional food applications. *S. thermophilus* ST4 powder (1 × 10^11^ CFU/g) was mixed at a 1:1 (w/w) ratio with sterile maltodextrin, producing a concentration of 1 × 10^10^ CFU/g intermediate powder. To prepare the high-dose suspension (1 × 10^9^ CFU/mL), 2 g of this powder was dissolved in 20 mL of sterile 0.9% saline. A concentration of 1 × 10^7^ CFU/mL was prepared by diluting the high-dose solution at a 1:99 (v/v) ratio and regarded as a low-dose suspension. Both doses were freshly prepared daily before use.

### 2.3 *In vitro* protein digestibility estimation

Enzymatic hydrolysis of ISP, which served as a pre-digestion step simulating gastrointestinal protein breakdown, was performed following the method of Zeng et al. (2013). A solution of 2.5% (w/v) soy protein was adjusted to pH 2 to simulate gastric conditions, followed by the addition of pepsin at 6.7% (w/v). Hydrolysis was carried out for 2 h at 37 °C. The pH was then adjusted to 7 to deactivate pepsin, and pancreatin was added for another 2 h of hydrolysis at 37 °C. The reaction was terminated by heat treatment to ensure enzyme inactivation, and the resulting hydrolysate was collected for further use.

*S. thermophilus* ST4 was incorporated into the hydrolysate obtained after the completion of enzymatic hydrolysis. *S. thermophilus* ST4 was added at a final concentration of 1 × 10^9^ CFU/mL and incubated for either 2 or 4 h to observe time-dependent effects on amino acid release. This step was done to examine whether the metabolic activity of *S. thermophilus* ST4 could further release amino acids from partially hydrolyzed protein through its intrinsic proteolytic system. Control without a probiotic was included. Samples were centrifuged (4,187 × g, 15 min) after incubation, filtered through 0.2 μm membranes for sterility, and stored at −20 °C for further measurement.

### 2.4 *In vivo* protein digestion assay

#### 2.4.1 Experimental design and animals

Our experiments were conducted with approval from the Institutional Animal Care and Use Committee (IACUC) of National Chung Hsing University (Nos. 111-092 and 112-122, on 12/08/2021 and 12/06/2023, respectively). The procedures followed institutional ethical standards and the 3Rs. Forty-eight 8-week-old male Sprague–Dawley rats were procured from BioLASCO Co., Taiwan, and maintained under standardized conditions (12-h light/dark cycle, 60% humidity, 22 ± 2 °C).

To reduce variability and ensure that serum amino acid profiles reflected dietary effects rather than developmental changes, all rats underwent a 4-week acclimation period. Afterward, animals (362.2–452.6 g) were categorized into eight weight classes and randomly allocated to one of six groups: three receiving a normal diet (ND) and three receiving a high-protein diet (HPD). ND groups were given standard chow (Rodent Diet 5001; 24.1% protein content), while HPD groups received a modified diet containing 43.3% protein content, achieved by blending chow with soy protein isolate at a 7:3 (w/w) ratio (see Supplementary Table S2).

Animals in both the ND and HPD dietary regimens were further divided into three groups. One group served as the control and received no probiotics, and the other two groups received either a low dose or a high dose of *S. thermophilus* ST4. Specifically, the three ND groups consisted of a control group (ND control), a low-dose group (ND_L_ST4), and a high-dose group (ND_H_ST4). Similarly, the three HPD groups included a control group without probiotic supplementation (HPD control), a low-dose group (HPD_L_ST4), and a high-dose group (HPD_H_ST4).

During the 2-week intervention, all groups received their assigned diets ad libitum. For probiotic-supplemented groups, 3 g of feed was blended with 1 mL of either 1 × 10^7^ CFU/mL or 1 × 10^9^ CFU/mL probiotic and molded into a pellet to ensure consistent intake. Pellets were given daily at 10:00 a.m., before feeding. Regular feed was provided only after confirming complete pellet consumption. Feed intake and body weight were recorded daily.

#### 2.4.2 Serum collection

After the intervention, rats underwent a 12-h fast to ensure that the serum amino acid profiles were not influenced by recent food intake, thereby reflecting the steady-state metabolic impact of the long-term probiotic intake under each diet condition. Blood (~0.2 mL) was collected using a sterile syringe and needle from the tail vein, then centrifuged at 2,000 × g for 10 min to obtain serum. Serum was stored at −20 °C until analysis.

### 2.5 Amino acid profile analysis

#### 2.5.1 Sample preparation

Serum and hydrolyzed ISP were prepared for subsequent analysis using a protocol based on Babu et al. (2002). Briefly, 5 μL of 2-mercaptoethanol was added to 0.1 mL of the sample and allowed to stand for 5 min at room temperature. Protein precipitation was induced by adding 395 μL of methanol under continuous mixing. Samples were subsequently chilled on ice for 15 min and centrifuged (4,187 × g, 15 min). Supernatants were filtered through 0.2 μm membranes.

#### 2.5.2 Amino acid measurement

After pre-treatment, samples were derivatized with o-phthalaldehyde (OPA) according to the protocol by Thermo Fisher Scientific Inc. (2012). In brief, 0.25 g of boric acid was dissolved in ultrapure water (9.5 mL), and the pH was set to 10.40 ± 0.02 by adding sodium hydroxide. To complete the buffer, 2-mercaptoethanol (0.02 mL) and Triton X-100 (0.03 mL) were added. Then, 5 mg of OPA crystals (P1378, Sigma-Aldrich, United States) were dissolved in methanol (0.1 mL) and mixed with the buffer to a volume of ~10 mL. Samples were combined with the OPA reagent in a 3:7 volume ratio.

A 32 μL aliquot of each derivatized sample was injected into a high-performance liquid chromatography (HPLC) system (Hitachi D-2000 HSM) equipped with a reverse-phase C18 column (InertSustain, 4.6 × 250 mm) and a fluorescence detector, following the method of Perucho et al. (2015). OPA-amino acid complexes were detected at an excitation and emission wavelength of 240 nm and 450 nm, respectively. The mobile phases included 1-h ultrasonically degassed solvent A (pH 5.6 sodium borate buffer (0.05 M) with 5% methanol) and solvent B (70% methanol). Gradient elution began at 25% B, increasing to 33% at 3 min, 45% at 4 min, 50% at 5 min, 55% at 10 min, 60% at 15 min, 70% at 22 min, 80% at 27 min, and 100% at 37 min. This was maintained for 10 min before re-equilibrating to 25% B over the final 5 min. The flow rate was maintained at 1 mL/min.

A standard mixture of 17 amino acids (AAS18, Supelco, United States), supplemented with glutamine (G3126, Sigma-Aldrich, United States) and asparagine (A0884, Sigma-Aldrich, United States), was used to identify peaks based on retention times. Individual amino acids were quantified, and total amino acid content was calculated as the sum of all amino acids.

### 2.6 Postprandial serum amino acid assay

A postprandial experiment was performed based on results from the long-term *in vivo* study. Ten adult Sprague–Dawley rats were used to assess the effect of *S. thermophilus* ST4 on serum essential amino acids throughout active protein digestion and absorption. After a 4-week acclimation period, all rats underwent a 12-h fast, and baseline blood samples were collected to determine initial amino acid concentrations. Animals with similar body weights (397.6–406.2 g) were then randomly divided into two HPD groups: a control (no probiotics) and a treatment group receiving *S. thermophilus* ST4 at 1 × 10^9^ CFU (AUC_ST4), with five rats per group.

The same probiotic suspension and pellet preparation used in the long-term study were applied. A pellet containing 1 mL of probiotic blended with 3 g of feed was prepared. Once the pellet was fully consumed, a timer was started to begin the postprandial phase. Blood was collected at 15 min, 1 h, 2 h, and 4 h to monitor serum amino acid changes and assess absorption following probiotic administration.

### 2.7 Statistical analysis

Treatment effects were evaluated using ANOVA in IBM SPSS Statistics (version 20; SPSS Inc., Chicago, IL, United States). In addition to comparing each probiotic dose with the corresponding control within each dietary group, a two-way ANOVA was performed for the *in vivo* study using a 2 × 3 factorial design. This analysis evaluated the main effects of diet type (normal vs. high-protein), probiotic dose (0, 1 × 10^7^, or 1 × 10^9^ CFU/day), and their interaction on serum amino acid concentrations. Scheffé’s *post hoc* test was applied to assess within- and between-group differences. Statistical significance was set at *p* < 0.05 or *p* < 0.01. Results are expressed as mean ± standard deviation (SD).

*In vitro* and *in vivo* experimental results were reported as percentage changes (%). The area under the curve (AUC) was measured using the linear trapezoidal method for postprandial amino acids, total amino acids (TAA), non-essential amino acids (NEAA), and essential amino acids (EAA) (Jäger et al., 2020), based on values at post-feeding time points.

## 3 Results

### 3.1 *Streptococcus thermophilus* ST4 ability in protein digestion

Compositional analysis of ISP identified glutamic acid (15.70 g/100 g) as the predominant amino acid, followed by aspartic acid (9.67 g/100 g) (Supplementary Table S3). Enzymatic hydrolysis resulted in a hydrolysis rate of approximately 47% based on total amino acid content in the hydrolysate relative to the original ISP.

As presented in Table 1, the incorporation of *S. thermophilus* ST4 significantly enhanced the levels of at least ten amino acids after both 2 and 4 h of incubation (*p* < 0.05). Four were essential amino acids (histidine, threonine, phenylalanine, and valine) exhibiting relative increases ranging from 30.1% to as high as 358.0%. Among them, histidine showed the greatest change in concentration. The cumulative effects of *S. thermophilus* ST4 on EAA, NEAA, and TAA at both time points are summarized in Table 2. Supplementation with *S. thermophilus* ST4 produced substantial enhancements (*p* < 0.01) of 69.1% in EAA, 124.4% in NEAA, and 96.9% in TAA.

**Table 1:** Changes in amino acid concentrations in hydrolyzed soy protein supernatant after *S. thermophilus* ST4 addition.

Amino acids	Control^a^	After the addition of *S. thermophilus* ST4^a^	
		2 h	4 h
Alanine	3.19 ± 0.12	5.94 ± 0.03^**^	7.01 ± 0.34^**#^
Arginine	1.26 ± 0.04	2.42 ± 0.15^**^	2.59 ± 0.08^**^
Aspartic acid	0.62 ± 0.03	1.44 ± 0.05^*^	1.74 ± 0.26^*^
Asparagine	2.38 ± 0.03	3.30 ± 0.33	3.38 ± 0.27
Cysteine	0.97 ± 0.44	1.69 ± 0.17	2.50 ± 0.23
Glutamic acid	0.41 ± 0.02	2.50 ± 0.20	2.02 ± 0.07
Glutamine	2.19 ± 0.09	5.25 ± 0.01^*^	6.99 ± 0.46^**^
Glycine	1.16 ± 0.29	1.51 ± 0.19	1.47 ± 0.21
Proline	1.33 ± 0.15	5.65 ± 0.26^**^	6.94 ± 0.32^**#^
Serine	0.16 ± 0.02	1.00 ± 0.14	1.42 ± 0.13^*^
Tyrosine	6.22 ± 0.81	9.12 ± 0.48	8.58 ± 0.49
Histidine^†^	1.19 ± 0.12	4.26 ± 0.04^**^	5.45 ± 0.28^**#^
Lysine^†^	2.15 ± 0.02	3.94 ± 0.07	3.21 ± 0.05
Threonine^†^	1.31 ± 0.19	2.58 ± 0.12^**^	2.64 ± 0.03^**^
Methionine^†^	0.77 ± 0.08	0.75 ± 0.02	0.68 ± 0.33
Phenylalanine^†^	5.75 ± 0.36	7.48 ± 0.29^*^	7.60 ± 0.45^*^
Leucine^†^	5.27 ± 0.05	4.82 ± 0.19	5.36 ± 0.30
Isoleucine^†^	1.17 ± 0.08	1.40 ± 0.25	2.51 ± 0.17
Valine^†^	1.33 ± 0.11	3.44 ± 0.04^**^	4.57 ± 0.43^**^

a Amino acid values are means ± SD expressed in g/100 g protein.

*Significant at *p* < 0.05 compared with control group.

**Significant at *p* < 0.01 compared with control group.

^#^Significant at *p* < 0.05 compared with 2 h group.

^†^Indicates an amino acid categorized as essential.

**Table 2:** Changes in EAA, NEAA, and TAA in hydrolyzed soy protein supernatant after addition of *S. thermophilus* ST4.

Amino acids	Control	After the addition of *S. thermophilus* ST4	
		2 h	4 h
Essential amino acid	18.94 ± 1.01	28.67 ± 1.02^**^	32.02 ± 2.04^**^
Non-essential amino acid	19.89 ± 2.04	39.82 ± 2.01^**^	44.64 ± 2.86^**^
Total amino acid	38.83 ± 3.05	68.49 ± 3.03^**^	76.46 ± 4.90^**^

Each value is the mean ± SD expressed in g/100 g protein.

*Significant at *p* < 0.05 compared with control group.

**Significant at *p* < 0.01 compared to control group.

### 3.2 Effect of *Streptococcus thermophilus* ST4 supplementation on *in vivo* serum amino acid levels

All animals remained active and healthy throughout the study. Following the 2-week intervention, no significant changes were observed in feed intake (+10.6% to +12.6%) or weight gain (+10.9% to +12.7%) across groups.

**Figure 1:**
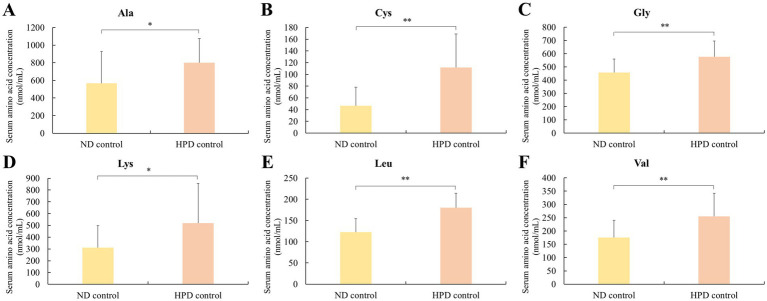
Effect of normal diet (ND) and high-protein diet (HPD) on serum amino acid concentrations in rats. Notable increases in the concentration of six serum amino acids (**A**: Alanine, **B**: Cysteine, **C**: Glycine, **D**: Lysine, **E**: Leucine, and **F**: Valine) were observed in the group of HPD control. Significant differences compared to the control group are indicated as *p* < 0.05 (^*^) and *p* < 0.01 (^**^). Data are expressed as mean ± SD (*n* = 8 per group).

A comparative assessment of serum amino acid composition revealed that a high-protein diet fed rats exhibited elevated levels of EAA (+32.7%) and TAA (+23.4%) relative to those on a normal diet (*p* < 0.05) (Table 3). This enhancement included substantial increases in specific amino acids such as alanine (+41.3%), cysteine (+140.5%), glycine (+26.5%), lysine (+66.4%), leucine (+47.0%), and valine (+45.0%), as illustrated in Figure 1. The complete dataset of quantified amino acids is provided in Supplementary Table S4 for reference.

**Table 3:** Effect of normal diet (ND) and high-protein diet (HPD) on serum EAA, NEAA, and TAA in rats.

Amino acids	ND control	HPD control
Essential amino acid	1164.87 ± 232.36	1545.38 ± 432.24^**^
Non-essential amino acid	2195.68 ± 573.63	2602.25 ± 652.49
Total amino acid	3360.56 ± 764.83	4147.64 ± 1029.31^*^

Each value is the mean ± SD expressed in nmol/mL of blood serum.

*Significant at *p* < 0.05 compared with ND control group.

**Significant at *p* < 0.01 compared with ND control group.

**Figure 2:**
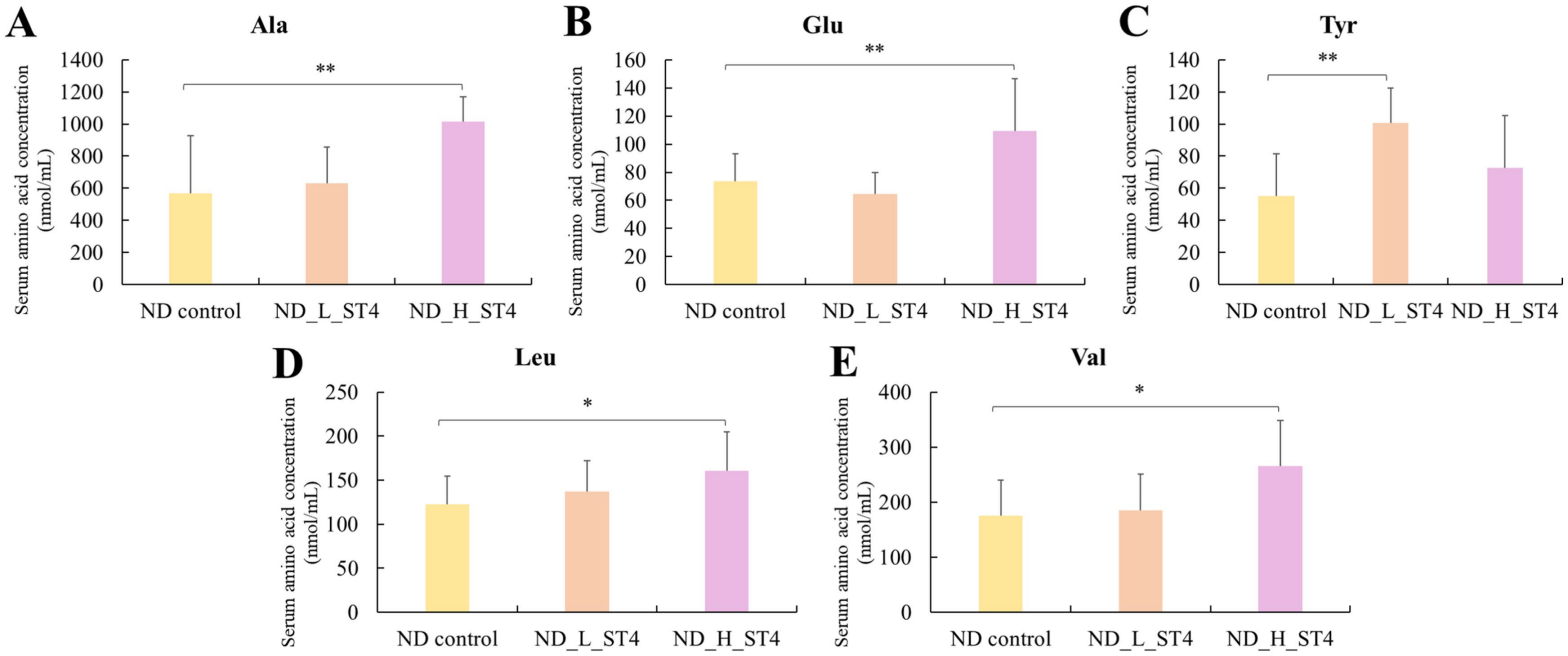
Effect of *Streptococcus thermophilus* ST4 on serum amino acid concentrations in rats under normal dietary conditions (ND). Notable increases in the concentration of five serum amino acids (**A**: Alanine, **B**: Glutamic acid, **C**: Tyrosine, **D**: Leucine, and **E**: Valine) were observed in the group of ND_L_ST4 (low-dose *S. thermophilus* ST4, 1 × 10^7^ CFU/day) and ND_H_ST4 (high-dose *S. thermophilus* ST4, 1 × 10^9^ CFU/day). Significant differences compared to the control group are indicated as *p* < 0.05 (^*^) and *p* < 0.01 (^**^). Data are expressed as mean ± SD (*n* = 8 per group).

As shown in Figure 2, the ND_L_ST4 group exhibited a significant increase in serum tyrosine levels (+81.7%, *p* < 0.05) compared to the ND control. In the high-dose group (1 × 10^9^ CFU/day), additional elevations were observed in alanine (+78.8%), glutamic acid (+31.0%), valine (+51.1%), and leucine (+38.9%) (*p* < 0.05). The observed values of each amino acid are available in Supplementary Table S5.

Table 4 summarizes the impacts of *S. thermophilus* ST4 on serum amino acids under a normal diet. While the low-dose group (1 × 10^7^ CFU/day) showed no significant changes, the high-dose group (ND_H_ST4) exhibited higher concentrations of EAA (+43.2%), NEAA (+26.6%), and TAA (+32.4%) than the ND control (*p* < 0.05).

**Table 4:** Effect of *S. thermophilus* ST4 on serum EAA, NEAA, and TAA in normal dietary group (ND) rats.

Amino acids	ND control	ND_L_ST4	ND_H_ST4
Essential amino acid	1164.87 ± 232.36	1337.88 ± 340.81	1667.72 ± 283.23^**^
Non-essential amino acid	2195.68 ± 573.63	2339.16 ± 598.47	2779.98 ± 291.66^*^
Total amino acid	3360.56 ± 764.83	3677.04 ± 845.97	4447.71 ± 438.82^**^

Each value is the mean ± SD expressed in nmol/mL of blood serum.

*Significant at *p* < 0.05 compared with ND control group.

**Significant at *p* < 0.01 compared with ND control group.

**Figure 3:**
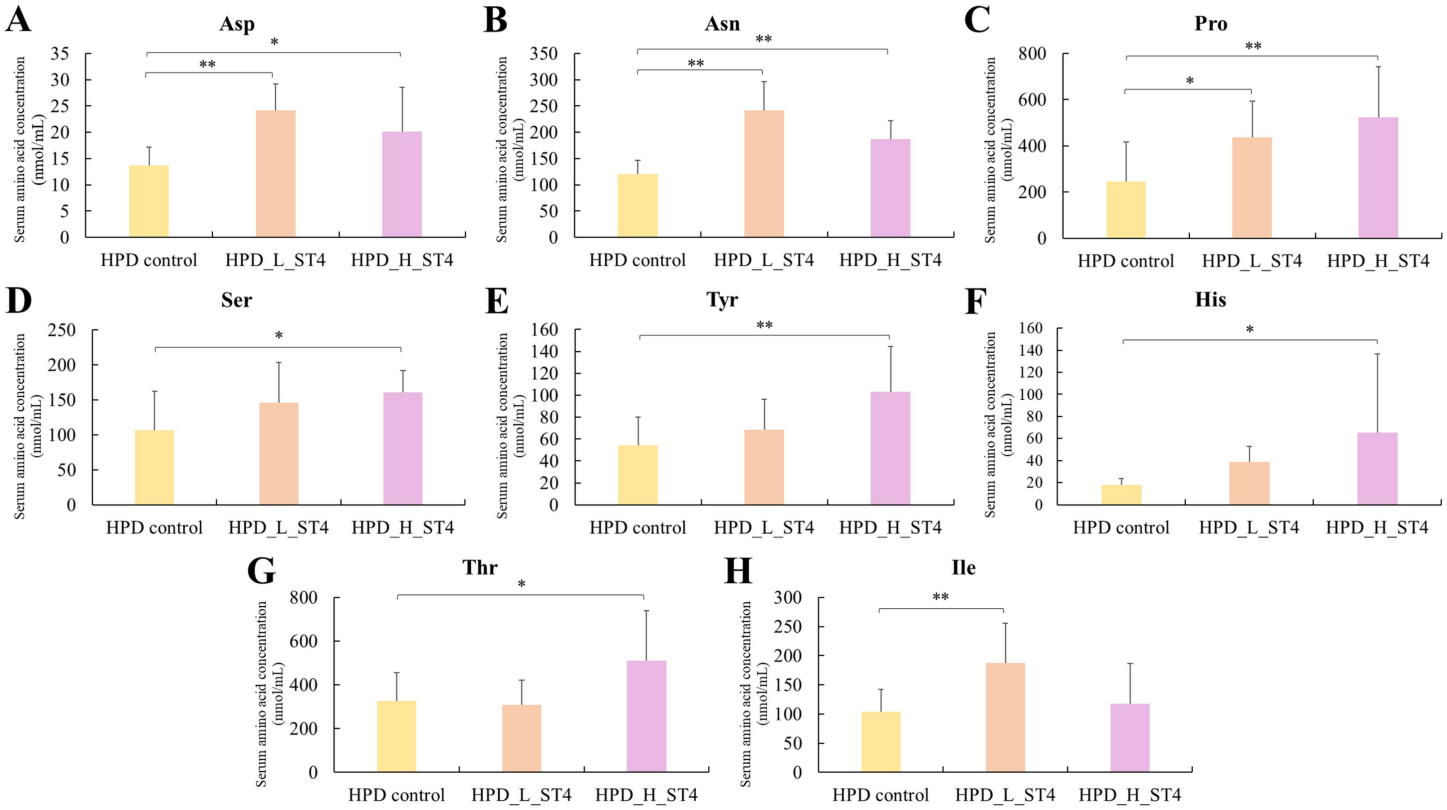
Effect of *Streptococcus thermophilus* ST4 on serum amino acid concentrations in rats under high-protein dietary conditions (HPD). Notable increases in the concentration of eight serum amino acids (**A**: Aspartic acid, **B**: Asparagine, **C**: Proline, **D**: Serine, **E**: Tyrosine, **F**: Histidine, **G**: Threonine, and **H**: Isoleucine) were observed in the group of HPD_L_ST4 (low-dose *S. thermophilus* ST4, 1 × 10^7^ CFU/day) and HPD_H_ST4 (high-dose *S. thermophilus* ST4, 1 × 10^9^ CFU/day). Significant differences compared to the control group are indicated as *p* < 0.05 (^*^) and *p* < 0.01 (^**^). Data are expressed as mean ± SD (*n* = 8 per group).

As shown in Figure 3, low-dose *S. thermophilus* ST4 supplementation (1 × 10^7^ CFU/day) resulted in notable elevations (*p* < 0.05) in asparagine (+100.3%), aspartic acid (+76.8%), proline (+78.8%), and isoleucine (+80.7%) relative to the control. In the HPD_H_ST4 group, further elevations were demonstrated in aspartic acid (+47.1%), asparagine (+55.3%), proline (+115.0%), serine (+51.2%), tyrosine (+89.5%), histidine (+259.1%), and threonine (+56.5%) (*p* < 0.05). The corresponding amino acid data are also presented in Supplementary Table S6.

Cumulative analysis in the high-protein diet groups revealed no significant differences in TAA, NEAA, or EAA of the HPD_L_ST4 group. In contrast, the HPD_H_ST4 group exhibited rises (*p* < 0.05) in TAA (+29.7%), NEAA (+25.0%), and EAA (+37.7%) relative to the HPD control (Table 5).

**Table 5:** Effect of *S. thermophilus* ST4 on serum EAA, NEAA, and TAA in high-protein dietary group (HPD) rats.

Amino acids	HPD control	HPD_L_ST4	HPD_H_ST4
Essential amino acid	1545.38 ± 432.24	1579.31 ± 103.27	2127.23 ± 400.52^**^
Non-essential amino acid	2602.25 ± 652.49	2735.38 ± 353.15	3253.55 ± 428.30^*^
Total amino acid	4147.64 ± 1029.31	4314.69 ± 321.61	5380.78 ± 513.01^**^

Each value is the mean ± SD expressed in nmol/mL of blood serum.

*Significant at *p* < 0.05 compared with HPD control group.

**Significant at *p* < 0.01 compared with HPD control group.

Factorial design analysis showed that diet type exerted significant effects on serum EAA, NEAA, and TAA levels, as well as on several individual amino acids, including aspartic acid, asparagine, cysteine, proline, valine, leucine, and lysine (*p* < 0.01), and phenylalanine and isoleucine (*p* < 0.05). The probiotic dose also significantly influenced the concentrations of EAA, NEAA, TAA, aspartic acid, asparagine, alanine, tyrosine, leucine, and threonine (*p* < 0.01), and affected the concentrations of glutamic acid, serine, valine, and lysine (*p* < 0.05). A significant diet-by-dose interaction was observed for aspartic acid, asparagine, tyrosine, and isoleucine (*p* < 0.01), as well as for glutamic acid, serine, alanine, glycine, arginine, proline, and histidine (*p* < 0.05; Supplementary Table S7).

### 3.3 Effect of *Streptococcus thermophilus* ST4 on postprandial serum amino acid

**Figure 4:**
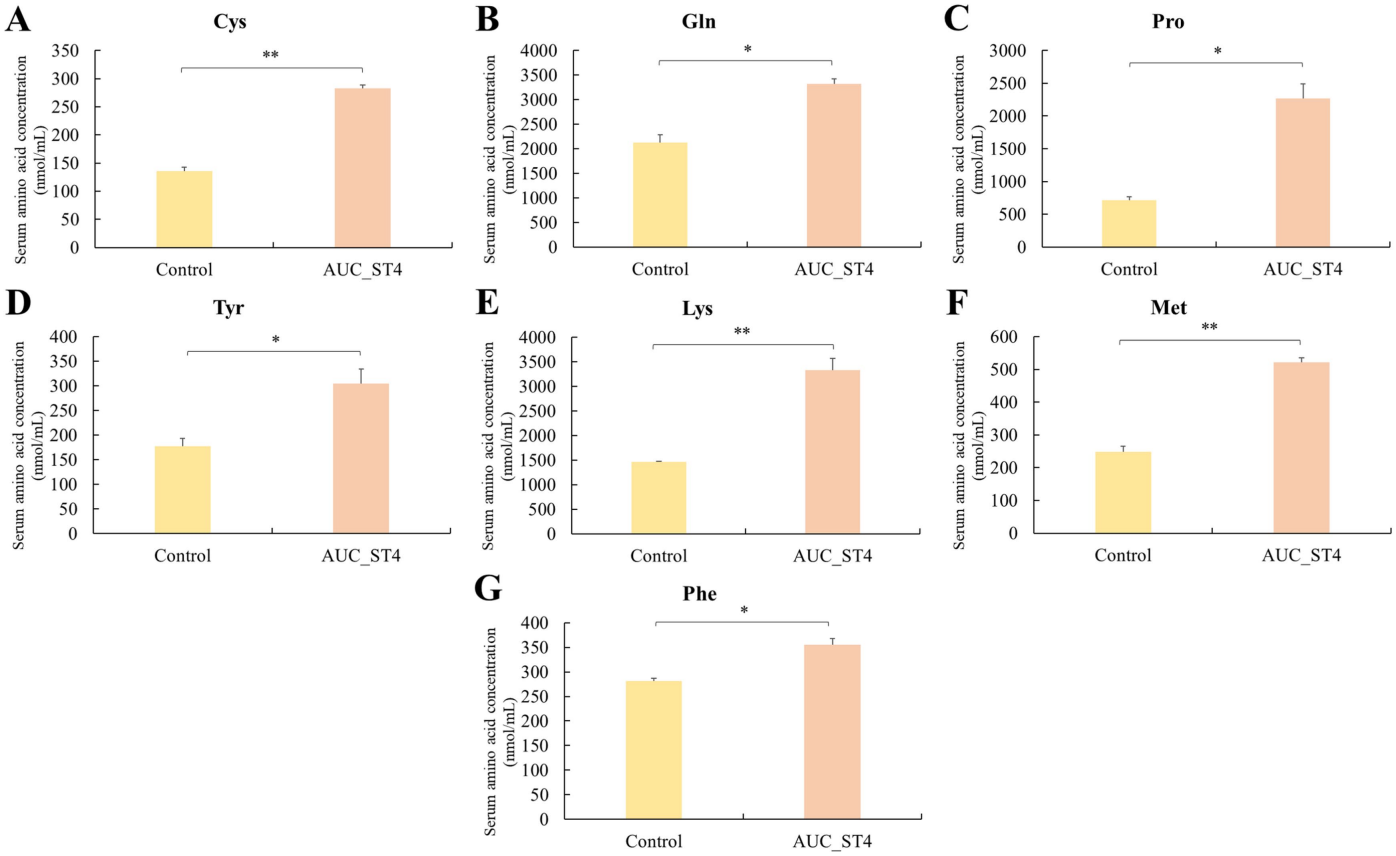
Comparison of amino acid absorption in postprandial serum following *Streptococcus thermophilus* ST4 supplementation. Notable increases in the area under the curve (AUC) of seven serum amino acids (**A**: Cysteine, **B**: Glutamine, **C**: Proline, **D**: Tyrosine, **E**: Lysine, **F**: Methionine, and **G**: Phenylalanine) were observed in the group of AUC_ST4 (1 × 10^9^ CFU, referred to as HPD_H_ST4). Significant differences compared to the control group are indicated as *p* < 0.05 (^*^) and *p* < 0.01 (^**^). Data are expressed as mean ± SD (*n* = 5 per group).

Seven amino acids exhibited significant elevations in the AUC of postprandial serum in the *S. thermophilus*-supplemented group, as depicted in Figure 4. The values were elevated (*p* < 0.05) for cysteine (+108.0%), glutamine (+56.4%), proline (+215.3%), tyrosine (+71.7%), lysine (+127.5%), methionine (+109.4%), and phenylalanine (+26.6%) in the AUC_ST4 group, with corresponding data provided in Supplementary Table S8.

To assess the overall effect of *S. thermophilus* ST4 on postprandial amino acid absorption in the postprandial model, Table 6 presents a summary of TAA, NEAA, and EAA changes, along with relevant statistical comparisons. The probiotic-supplemented group showed significant increases (*p* < 0.01) in TAA values (+59.7%), NEAA (+55.5%), and EAA (+54.2%) compared to the control.

**Table 6:** Comparison of the area under the curve AUC [nmol/mL • 240 min] of postprandial EAA, NEAA, and TAA.

Amino acids	Control	AUC_ST4
Essential amino acid	5247.50 ± 2.62	8092.65 ± 378.70^**^
Non-essential amino acid	10925.96 ± 101.91	16995.21 ± 402.87^**^
Total amino acid	16177.93 ± 110.85	25839.58 ± 1087.27^**^

Each value is presented as mean ± standard deviation (SD).

*Significant at *p* < 0.05 compared with control group.

**Significant at *p* < 0.01 compared with control group.

## 4 Discussion

An initial evaluation to determine whether *S. thermophilus* ST4 could enhance protein digestion was performed using *in vitro* digestion assays. The observed elevation in amino acid concentrations aligns with earlier findings demonstrating improved soy protein hydrolysis by probiotic strains, evidenced by elevated free α-amino nitrogen concentrations following simulated digestion when compared to a probiotic-free control (Marttinen et al., 2023). Probiotics are known to facilitate amino acid release and metabolism through enzymatic transformation and translocation pathways. These processes are mediated by three primary transport systems, including proton motive force-dependent permeases (PTRs), ATP-driven ATP-binding cassette (ABC) transporters, and reverse transporters that function along concentration gradients (Savijoki et al., 2006).

Comparable findings were reported for *Bacillus coagulans* BC30. This strain enhanced *in vitro* soy protein digestion by increasing the release of short peptides and amino acids and thereby elevating α-amino nitrogen and total nitrogen levels (Keller et al., 2017). In this study, *S. thermophilus* ST4 likewise promoted essential amino acid release from hydrolyzed soy protein, suggesting its proteolytic involvement and supporting further *in vivo* investigation.

Building upon these observations, the *in vivo* experiments incorporated ISP with *S. thermophilus* ST4 supplementation to assess its effects on circulating amino acid concentrations in a rat model. The observed changes in serum amino acid composition may be attributed to the metabolic activity of *S. thermophilus* ST4. This was supported by Rodríguez-Serrano et al. (2018), who reported that *S. thermophilus* possesses a specialized proteolytic system comprising 14 distinct peptidases. Additionally, Boulay et al. (2020) identified a proteome of 328 proteins in *S. thermophilus* LMD-9 when cultured in soybean milk, of which 63 proteins (19.2%) were involved in protein transport and metabolism.

Elevated serum levels of TAA, NEAA, and EAA by high-protein diet intervention suggest that circulating amino acids can be directly enhanced through increased protein intake. This effect appeared to result from the upregulation of digestive enzyme activity, as well as greater substrate input (Tobón-Cornejo et al., 2025). Notably, probiotic supplementation led to a measurable alteration of long-term serum amino acids under a normal diet regimen. This indicated that the probiotics promoted amino acid bioavailability even in the absence of elevated protein intake through improved digestion and intestinal absorption of protein-derived amino acids (Carneiro et al., 2023). These outcomes implied that probiotic interventions were able to confer additional metabolic benefits under standard dietary conditions.

Liu et al. (2022) reported that *Limosilactobacillus reuteri* DSM 17938 increased plasma amino acids and related metabolites in mice, highlighting that such effects were strain-specific rather than universal across probiotics. Similarly, probiotics significantly elevated branched-chain amino acids, EAA, and TAA levels in humans as found by Jäger et al. (2020), corroborating our findings. In another *in vivo* study, *Lactiplantibacillus plantarum* increased serum lysine and methionine in high-protein-fed rats (Wibisono et al., 2025), whereas this was not observed in *S. thermophilus* ST4. *S. thermophilus* ST4 elevated histidine and isoleucine, suggesting a unique species-specific functional profile that may complement other strains. Evidence indicates that probiotics metabolically transform plant proteins, facilitating macronutrient interconversion and generating bioactives. These processes facilitate digestion, nutrient absorption, and help overcome the plant protein limitations (Wu et al., 2024).

Our results revealed that low-dose *S. thermophilus* ST4 (1 × 10^7^ CFU/day) primarily elevated NEAA levels, whereas the high dose (1 × 10^9^ CFU/day) prominently increased several EAAs, including leucine, valine, histidine, threonine, and isoleucine. These effects likely reflect enhanced hydrolysis or facilitated amino acid absorption.

The results of the factorial analysis demonstrated that dietary protein load and *S. thermophilus* ST4 supplementation applied alone or in combination shaped circulating amino acid profiles and highlighted their complementary roles in modulating amino acid metabolism. The strong influence of diet type on multiple EAAs and NEAAs aligned with the well-established impact of high-protein intake on amino acid absorption. In contrast, the probiotic dose affected a partially overlapping but distinct set of amino acids, suggesting that *S. thermophilus* ST4 facilitated the liberation and intestinal transport of amino acids, rather than simply mirroring dietary effects. The significant diet-probiotic dose interaction also indicated that the metabolic contribution of *S. thermophilus* ST4 was amplified under high-protein conditions for several amino acids, particularly aspartic acid, asparagine, tyrosine, and isoleucine.

To validate whether probiotic supplementation could influence serum amino acid concentrations during active digestion, a postprandial experimental model was incorporated. The high-protein diet combined with *S. thermophilus* ST4 at 1 × 10^9^ CFU was selected for this analysis, as it elicited a more substantial increase in serum amino acid levels compared to the lower probiotic dose and the normal diet regimen in the preceding long-term *in vivo* study. Serum amino acid levels were monitored over the first 4 h following *S. thermophilus* ST4 administration, and AUC was calculated for comparison. A marked rise in total amino acids during this period suggested enhanced intestinal absorption, which may contribute to better fulfillment of dietary amino acid requirements.

This observation was consistent with some previous reports indicating that *S. thermophilus* efficiently consumes and metabolizes several amino acids, including lysine, glutamine, methionine, tyrosine, and cysteine, during its growth cycle, underscoring its role in nitrogen metabolism and amino acid turnover (Hong et al., 2015). The expression of specific proteolytic enzymes, such as aminopeptidases like PepN, showed high activity toward hydrophobic and basic amino acids (e.g., lysine and phenylalanine). This activity could lead to the increased bioavailability *in vivo* of these amino acids (Rul et al., 1994).

Jäger et al. (2020) reported that co-administration of *Lacticaseibacillus paracasei* strains LPC-S01 and LP-DG with pea protein elevated the AUC of tyrosine, histidine, methionine, valine, leucine, isoleucine, and total EAAs. The combination of plant-based protein with BC30 was also observed by Walden et al. (2024) to elevate AUC values of cysteine, EAAs, and total amino acids in healthy females. Our short-term model demonstrated that *S. thermophilus* ST4 can effectively enhance circulating amino acid concentrations when administered alongside plant-derived protein.

Furthermore, *S. thermophilus* ST4 supplementation in a high-protein diet resulted in improved serum levels of key EAAs, particularly lysine, methionine, and phenylalanine. These enhancements directly addressed the nutritional limitations associated with plant-based proteins, especially their low methionine content compared to animal protein, and contributed to a more complete amino acid profile and improved bioavailability (Phillips, 2016). These results emphasize the potential role of *S. thermophilus* ST4 in enhancing acute protein absorption efficiency, strengthening its application as a functional dietary adjunct in plant-based consumption patterns.

## 5 Conclusion

Our study demonstrated that *S. thermophilus* ST4 effectively enhanced *in vitro* plant protein digestion and increased amino acid content. This was confirmed *in vivo*, where co-administration with soy protein elevated essential amino acid levels and the postprandial profile of serum amino acids. These findings highlight the potency of *S. thermophilus* ST4 to assist the strategic development of plant-derived protein enhancement. Future clinical studies are warranted to evaluate its metabolic outcomes in human populations.

## Data Availability

The original contributions presented in the study are included in the article/Supplementary material, further inquiries can be directed to the corresponding author.
