# Supplementary material for: Enhancing essential amino acid bioavailability of soy protein through Streptococcus thermophilus ST4 supplementation
Source: Front Microbiol. 2026 Jan 6;16:1740277. doi: 10.3389/fmicb.2025.1740277 (PMC12816350; doi:10.3389/fmicb.2025.1740277)
Supplement: Supplementary file 1 [file Table_1.docx]

Supplementary Material

(A)

| Primer | Sequence | Reference |
| --- | --- | --- |
| fD1 | 5’-AGAGTTTGATCCTGGCTCAG-3’ | (Weisburg et al., 1991) |
| rP1 | 5’-ACGGTTACCTTGTTACGACTT-3’ | (Weisburg et al., 1991) |

(B)

CTTCTTGGATGAGTTGCGAACGGGTGAGTAACGCGTAGGTAACCTGCCTTGTAGCGGGGGATAACTATTGGAAACGATAGCTAATACCGCATAACAATGGATGACACATGTCATTTATTTGAAAGGGGCAATTGCTCCACTACAAGATGGACCTGCGTTGTATTAGCTAGTAGGTGAGGTAATGGCTCACCTAGGCGACGATACATAGCCGACCTGAGAGGGTGATCGGCCACACTGGGACTGAGACACGGCCCAGACTCCTACGGGAGGCAGCAGTAGGGAATCTTCGGCAATGGGGGCAACCCTGACCGAGCAACGCCGCGTGAGTGAAGAAGGTTTTCGGATCGTAAAGCTCTGTTGTAAGTCAAGAACGGGTGTGAGAGTGGAAAGTTCACACTGTGACGGTAGCTTACCAGAAAGGGACGGCTAACTACGTGCCAGCAGCCGCGGTAATACGTAGGTCCCGAGCGTTGTCCGGATTTATTGGGCGTAAAGCGAGCGCAGGCGGTTTGATAAGTCTGAAGTTAAAGGCTGTGGCTCAACCATAGTTCGCTTTGGAAACTGTCAAACTTGAGTGCAGAAGGGGAGAGTGGAATTCCATGTGTAGCGGTGAAATGCGTAGATATATGGAGGAACACCGGTGGCGAAAGCGGCTCTCTGGTCTGTAACTGACGCTGAGGCTCGAAAGCGTGGGGAGCGAACAGGATTAGATACCCTGGTAGTCCACGCCGTAAACGATGAGTGCTAGGTGTTGGATCCTTTCCGGGATTCAGTGCCGAAGCTAACGCATTAAGCACTCCGCCTGGGGAGTACGACCGCAAGGTTGAAACTCAAAGGAATTGACGGGGGCCCGCACAAGCGGTGGAGCATGTGGTTTAATTCGAAGCAACGCGAAGAACCTTACCAGGTCTTGACATCCCGATGCTATTTCTAGAGATAGAAAGTTACTTCGGTACATCGG

(C)


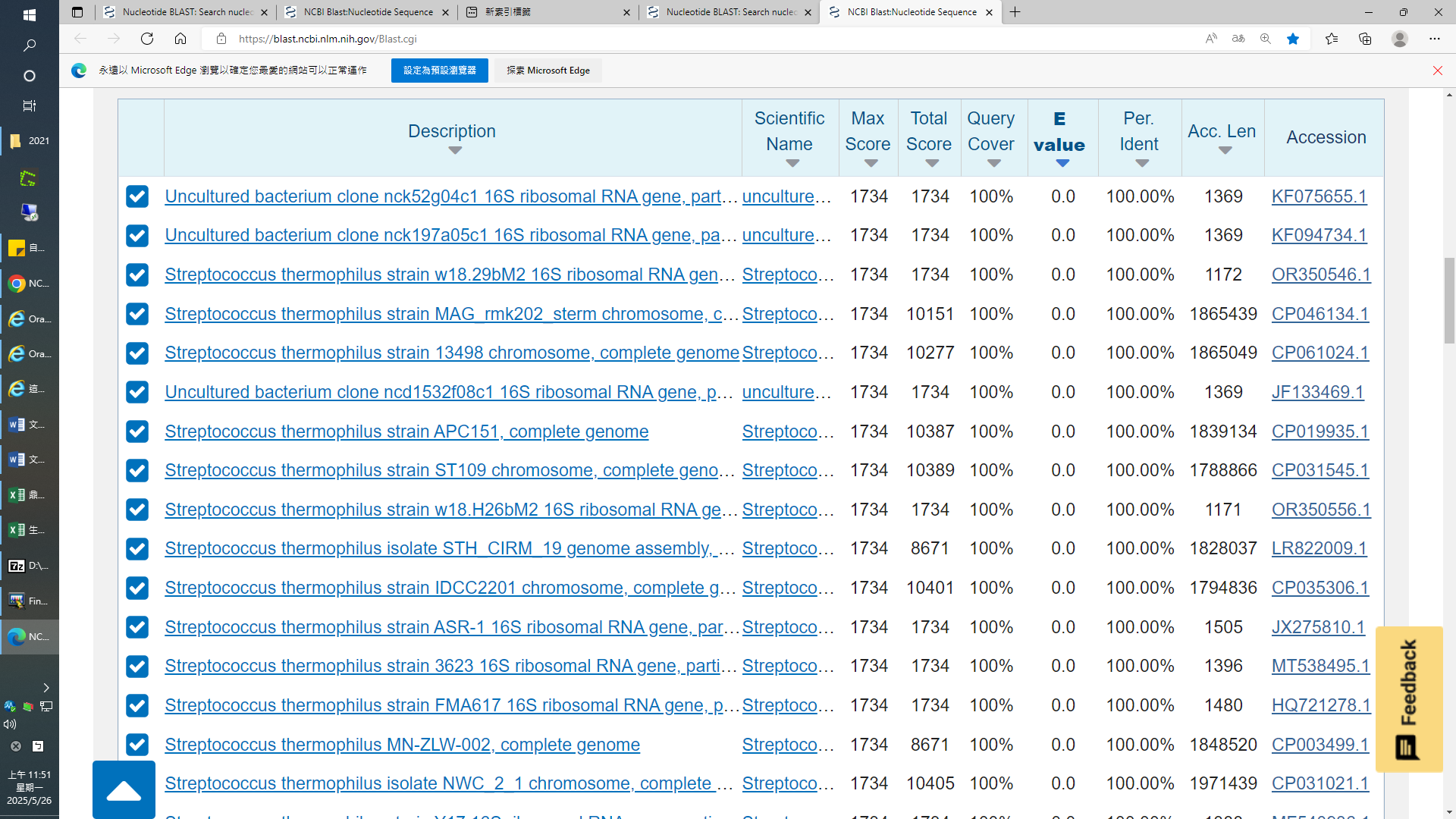


**Supplementary Figure S1.** Bacterial identification of *Streptococcus thermophilus* ST4*.* (A) Primers used for 16S rRNA gene analysis, (B) partial sequence of 16S rRNA gene, and (C) top-listed BLAST results.

**Description of Figure S1**

The S. thermophilus ST4 strain used in this study was originally isolated from raw bovine milk.

The 16S rRNA gene was amplified by PCR using primers listed in Supplementary Figure S1 (A), referenced according to Weisburg et al. (1991). PCR products were subjected to Sanger sequencing, and the sequence quality was assessed by Mission Biotech Co., Ltd. (Taipei, Taiwan) using the Applied Biosystems 3730xl DNA Analyzer. The finalized 16S rRNA gene sequence and the corresponding BLASTn alignment results are provided in Supplementary Figure S1 (B) and (C), respectively.

The ST4 strain has been deposited in the Bioresource Collection and Research Center, Food Industry Research and Development Institute, Taiwan (BCRC 910922), and the Deutsche Sammlung von Mikroorganismen und Zellkulturen (DSM 33165), Germany.

ST4 was cultured in broth consisting of 4% glucose, 2.5% peptone, 0.5% Sodium acetate, 0.4% Sodium citrate, and 0.01% Magnesium sulfate at 37°C for 15~24 h. After centrifugation, the bacterial mass was harvested and freeze-dried for functional testing.


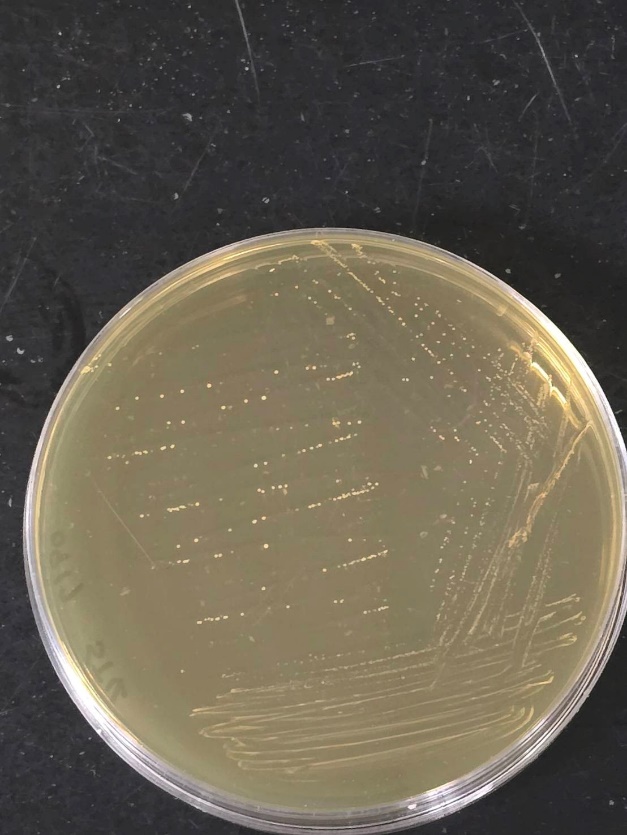

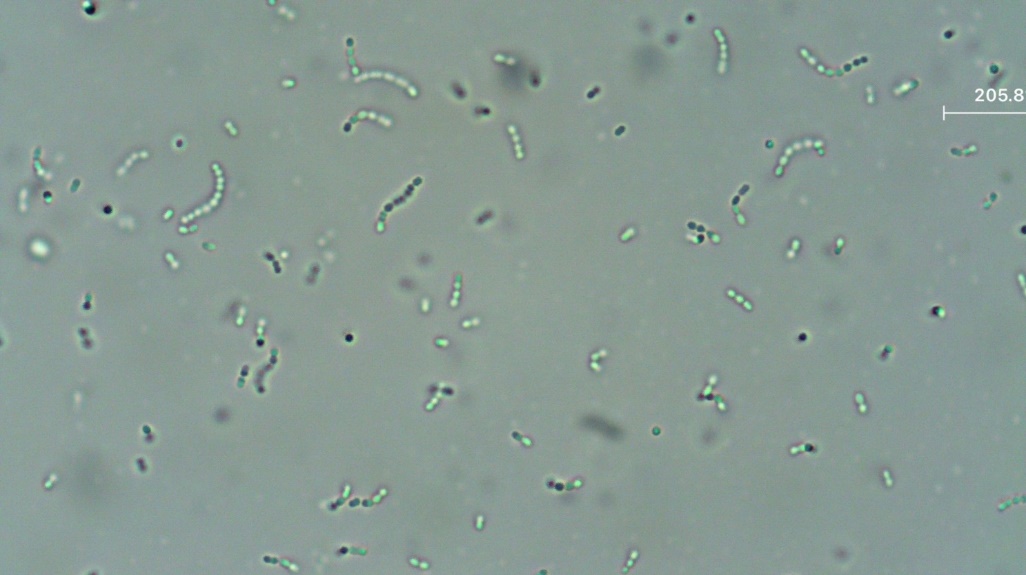


**Supplementary Figure S2.** Morphological characteristic and microscopic appearance of *Streptococcus thermophilus* ST4.

**Description of Figure S2**

*Streptococcus thermophilus* ST4 displayed smooth, milky-white colonies with well-defined, regular margins when cultured on MRS agar, and exhibited a convex elevation when viewed from the side. Microscopic examination revealed that ST4 cells were spherical or ovoid in shape and typically appeared in chains. This strain is a Gram-positive coccus, negative for both catalase and oxidase activity, non-motile, and capable of growing under both aerobic and anaerobic conditions. No endospore formation was observed.

**Supplementary Table S1.** Acid‐tolerance of *Streptococcus thermophilus* ST4.

| Strain | Initial count (0 h) | Count after 3 h | Survival rate (%) |
| --- | --- | --- | --- |
| ST4 | 5.0 × 10^7^ CFU | 3.0 × 10^6^ CFU | 84 |

**Description of Table S1**

*S. thermophilus* ST4 was exposed to simulated gastric fluid (pH 2.0) for 3 h. Viability was assessed by comparing CFU counts at 0 h and 3 h, and expressed as percent survival. *S. thermophilus* ST4 showed a 16% reduction.

**Supplementary Table S2.** Composition of standard chow diet and high-protein diet.

| Ingredient (g/100 g diet) | Standard chow diet | High-protein diet |
| --- | --- | --- |
| LabDiet 5001^1^ | 100.0 | 70.0 |
| Soy protein isolate | -- | 30.0 |
| Composition |  |  |
| Protein | 24.1 | 43.3 |
| Alanine | 1.4 | 2.1 |
| Arginine | 1.5 | 3.0 |
| Aspartic acid | 2.7 | 4.8 |
| Cysteine | 0.4 | 0.6 |
| Glutamic acid | 4.5 | 7.9 |
| Glycine | 1.2 | 1.9 |
| Histidine^†^ | 0.6 | 1.1 |
| Isoleucine^†^ | 1.0 | 1.9 |
| Leucine^†^ | 1.8 | 3.3 |
| Lysine^†^ | 1.4 | 2.4 |
| Methionine^†^ | 0.6 | 0.7 |
| Phenylalanine^†^ | 1.1 | 2.0 |
| Proline | 1.4 | 2.2 |
| Serine | 1.1 | 2.1 |
| Threonine^†^ | 0.9 | 1.6 |
| Tyrosine | 0.7 | 1.5 |
| Valine^†^ | 1.1 | 2.0 |
| Fat | 5.1 | 4.5 |
| Carbohydrate | 48.1 | 33.7 |
| Energy^2^ (kcal/g) | 3.35 | 3.49 |

^1^ Laboratory Rodent Diet 5001 (in powder): LabDiet® (2022).

^2^ *Energy* (kcal/g) = (Protein% × 4 + Fat% × 9 + Carbohydrate% × 4)/100.

^†^ Indicates an amino acid categorized as essential.

**Description of Table S2**

Laboratory Rodent Diet 5001 Ingredients: Ground corn, dehulled soybean meal, dried plain beet pulp, fish meal, ground oats, dehydrated alfalfa meal, brewers dried yeast, cane molasses, wheat germ, dried whey, porcine animal fat preserved with BHA and citric acid, porcine meat and bone meal, wheat middlings, salt, Calcium carbonate, DL-Methionine, Choline chloride, Cholecalciferol (Vitamin D3), Vitamin A acetate, Folic acid, Menadione dimethylpyrimidinol bisulfite (Vitamin K), Pyridoxine hydrochloride, Thiamine mononitrate, Nicotinic acid, Calcium pantothenate, DL-Alpha Tocopheryl acetate (Vitamin E), Manganous oxide, Vitamin B12 supplement, Zinc oxide, Ferrous carbonate, Copper sulfate, Ferrous sulfate, Riboflavin supplement, Zinc sulfate, Calcium iodate, Cobalt carbonate, Biotin, and Sodium selenite (LabDiet®, 2022).

**Supplementary Table S3.** Amino acid content in soy protein isolate (ISP).

| Amino acids | Content (g/100 g) |
| --- | --- |
| Alanine | 3.56 ± 0.32 |
| Arginine | 6.34 ± 0.55 |
| Aspartic acid | 9.67 ± 0.82 |
| Cysteine | 1.05 ± 0.09 |
| Glutamic acid | 15.70 ± 1.32 |
| Glycine | 3.57 ± 0.29 |
| Histidine^†^ | 2.18 ± 0.17 |
| Isoleucine^†^ | 4.06 ± 0.31 |
| Leucine^†^ | 6.66 ± 0.54 |
| Lysine^†^ | 4.73 ± 0.21 |
| Methionine^†^ | 1.06 ± 0.09 |
| Phenylalanine^†^ | 4.29 ± 0.35 |
| Proline | 4.04 ± 0.27 |
| Serine | 4.29 ± 0.34 |
| Threonine^†^ | 3.15 ± 0.25 |
| Tyrosine | 3.18 ± 0.27 |
| Valine^†^ | 4.12 ± 0.30 |
| Essential amino acid | 31.40 ± 2.34 |
| Non-essential amino acid | 51.41 ± 4.26 |
| Total amino acid | 82.81 ± 6.60 |

Each data is presented as mean ± standard deviation (SD).

^†^ Indicates an amino acid categorized as essential.

**Description of Table S3**

For the determination of the amino acid composition of ISP, a chemical hydrolysis method was performed as described by Henderson et al. (2000) to obtain the hydrolysate containing free amino acids. Approximately 0.1 g of ISP was hydrolyzed in 40 mL of 6 N hydrochloric acid for 24 h at 110°C. A suitable amount of phenol was added to prevent tyrosine from becoming halogenated.

**Supplementary Table S4.** Effects of normal diet and high-protein diet on serum amino acid concentrations.

| Amino acids | ND control^a^ | HPD control^a^ |
| --- | --- | --- |
| Alanine | 567.14 ± 360.71 | 801.58 ± 273.69^*^ |
| Arginine | 95.69 ± 25.52 | 80.29 ± 19.38 |
| Aspartic acid | 14.17 ± 3.68 | 13.68 ± 3.50 |
| Asparagine | 172.64 ± 55.33 | 120.77 ± 26.34^**^ |
| Cysteine | 46.55 ± 31.54 | 111.94 ± 57.18^**^ |
| Glutamic acid | 73.75 ± 19.47 | 79.04 ± 37.97 |
| Glutamine | 374.35 ± 88.80 | 412.63 ± 221.15 |
| Glycine | 456.70 ± 103.16 | 577.66 ± 117.61^**^ |
| Proline | 204.80 ± 240.40 | 243.97 ± 173.85 |
| Serine | 134.52 ± 56.95 | 106.26 ± 56.41 |
| Tyrosine | 55.32 ± 26.08 | 54.38 ± 25.60 |
| Histidine^†^ | 30.50 ± 24.30 | 18.24 ± 5.37 |
| Lysine^†^ | 312.26 ± 186.27 | 519.47 ± 337.46^*^ |
| Threonine^†^ | 275.52 ± 156.06 | 326.92 ± 128.27 |
| Methionine^†^ | 95.93 ± 34.71 | 87.12 ± 52.21 |
| Phenylalanine^†^ | 42.73 ± 16.87 | 54.66 ± 24.48 |
| Leucine^†^ | 122.52 ± 32.24 | 180.13 ± 33.84^**^ |
| Isoleucine^†^ | 109.43 ± 43.30 | 103.58 ± 38.83 |
| Valine^†^ | 175.96 ± 64.43 | 255.22 ± 87.07^**^ |

^a^ Amino acid values are means ± SD expressed in nmol/mL of serum.

^*^ Significant at *p* < 0.05; ^**^significant at *p* < 0.01 compared with ND control group.

^†^ Indicates an amino acid categorized as essential.

**Supplementary Table S5.** Effects of *Streptococcus thermophilus* ST4 on serum amino acid concentrations in normal dietary group (ND) rats.

| Amino acids | ND control^a^ | ND_L_ST4^a^ | ND_H_ST4^a^ |
| --- | --- | --- | --- |
| Alanine | 567.14 ± 360.71 | 632.31 ± 224.87 | 1014.31 ± 153.40^**#^ |
| Arginine | 95.69 ± 25.52 | 61.44 ± 24.37^**^ | 92.11 ± 26.14^#^ |
| Aspartic acid | 14.17 ± 3.68 | 12.18 ± 3.30 | 17.85 ± 5.96^#^ |
| Asparagine | 172.64 ± 55.33 | 121.22 ± 13.36^*^ | 168.48 ± 40.27 |
| Cysteine | 46.55 ± 31.54 | 67.31 ± 41.43 | 59.83 ± 35.08 |
| Glutamic acid | 73.75 ± 19.47 | 64.56 ± 15.22 | 109.37 ± 37.25^**##^ |
| Glutamine | 374.35 ± 88.80 | 389.77 ± 167.77 | 429.63 ± 103.74 |
| Glycine | 456.70 ± 103.16 | 453.80 ± 178.09 | 517.52 ± 70.82 |
| Proline | 204.80 ± 240.40 | 342.96 ± 270.93 | 144.13 ± 61.34 |
| Serine | 134.52 ± 56.95 | 93.05 ± 41.87 | 154.05 ± 28.06^#^ |
| Tyrosine | 55.32 ± 26.08 | 100.50 ± 21.87^**^ | 72.66 ± 32.68 |
| Histidine^†^ | 30.50 ± 24.30 | 49.43 ± 30.45 | 29.10 ± 12.38 |
| Lysine^†^ | 312.26 ± 186.27 | 442.41 ± 222.76 | 479.33 ± 128.32 |
| Threonine^†^ | 275.52 ± 156.06 | 346.47 ± 243.99 | 454.14 ± 187.94 |
| Methionine^†^ | 95.93 ± 34.71 | 73.78 ± 27.90 | 98.25 ± 24.76 |
| Phenylalanine^†^ | 42.73 ± 16.87 | 28.33 ± 24.18 | 38.46 ± 20.93 |
| Leucine^†^ | 122.52 ± 32.24 | 137.04 ± 35.03 | 160.49 ± 43.99^*^ |
| Isoleucine^†^ | 109.43 ± 43.30 | 74.73 ± 30.30 | 142.09 ± 51.87^##^ |
| Valine^†^ | 175.96 ± 64.43 | 185.64 ± 65.85 | 265.83 ± 82.02^*#^ |

^a^ Amino acid values are means ± SD expressed in nmol/mL of serum.

^*^ Significant at *p* < 0.05; ^**^significant at *p* < 0.01 compared with ND control group.

^#^ Significant at *p* < 0.05; ^##^significant at *p* < 0.01 compared with low-dose ST4 treatment.

^†^ Indicates an amino acid categorized as essential.

**Supplementary Table S6.** Effects of *Streptococcus thermophilus* ST4 on serum amino acid concentrations in high-protein dietary group (HPD) rats.

| Amino acids | HPD control^a^ | HPD_L_ST4^a^ | HPD_H_ST4^a^ |
| --- | --- | --- | --- |
| Alanine | 801.58 ± 273.69 | 728.84 ± 59.58 | 881.43 ± 89.71 |
| Arginine | 80.29 ± 19.38 | 93.34 ± 36.46 | 111.72 ± 47.97 |
| Aspartic acid | 13.68 ± 3.50 | 24.19 ± 4.96^**^ | 20.13 ± 8.40^*^ |
| Asparagine | 120.77 ± 26.34 | 241.95 ± 53.90^**^ | 187.56 ± 34.31^**##^ |
| Cysteine | 111.94 ± 57.18 | 105.31 ± 85.05 | 114.63 ± 76.25 |
| Glutamic acid | 79.04 ± 37.97 | 115.37 ± 65.02 | 98.12 ± 29.32 |
| Glutamine | 412.63 ± 221.15 | 382.56 ± 118.92 | 524.35 ± 143.41 |
| Glycine | 577.66 ± 117.61 | 392.82 ± 108.33^**^ | 527.42 ± 116.10^#^ |
| Proline | 243.97 ± 173.85 | 436.21 ± 156.71^*^ | 524.42 ± 217.20^**^ |
| Serine | 106.26 ± 56.41 | 146.05 ± 57.09 | 160.65 ± 30.82^*^ |
| Tyrosine | 54.38 ± 25.60 | 68.69 ± 27.65 | 103.05 ± 41.58^**^ |
| Histidine^†^ | 18.24 ± 5.37 | 38.95 ± 14.03 | 65.50 ± 71.08^*^ |
| Lysine^†^ | 519.47 ± 337.46 | 450.60 ± 81.46 | 724.59 ± 287.36 |
| Threonine^†^ | 326.92 ± 128.27 | 307.91 ± 113.35 | 511.61 ± 228.52^*#^ |
| Methionine^†^ | 87.12 ± 52.21 | 80.52 ± 22.24 | 129.61 ± 44.38 |
| Phenylalanine^†^ | 54.66 ± 24.48 | 40.22 ± 22.95 | 51.47 ± 14.48 |
| Leucine^†^ | 180.13 ± 33.84 | 198.47 ± 32.87 | 215.44 ± 70.04 |
| Isoleucine^†^ | 103.58 ± 38.83 | 187.18 ± 68.73^**^ | 117.60 ± 68.42^#^ |
| Valine^†^ | 255.22 ± 87.07 | 275.41 ± 79.80 | 311.38 ± 131.53 |

^a^ Amino acid values are means ± SD expressed in nmol/mL of blood serum.

^*^ Significant at *p* < 0.05; ^**^significant at *p* < 0.01 compared with HPD control group.

^#^ Significant at *p* < 0.05; ^##^significant at *p* < 0.01 compared with low-dose ST4 treatment.

^†^ Indicates an amino acid categorized as essential.

**Supplementary Table S7.** Two-way ANOVA summary.

| Outcome | F_diet | *p*_diet | Sig_diet | F_dose | *p*_dose | Sig_dose | F_interaction | *p*_interaction | Sig_interaction |
| --- | --- | --- | --- | --- | --- | --- | --- | --- | --- |
| TAA | 19.327 | 0.000** | 0.010 | 15.715 | 0.000** | 0.010 | 0.196 | 0.822^ns^ | 0.050 |
| NEAA | 11.359 | 0.001** | 0.010 | 8.890 | 0.000** | 0.010 | 0.034 | 0.967^ns^ | 0.050 |
| EAA | 21.456 | 0.000** | 0.010 | 18.245 | 0.000** | 0.010 | 0.584 | 0.560^ns^ | 0.050 |
| Alanine | 1.272 | 0.263^ns^ | 0.050 | 8.596 | 0.000** | 0.010 | 3.565 | 0.034* | 0.050 |
| Arginine | 2.826 | 0.097^ns^ | 0.050 | 3.400 | 0.039* | 0.050 | 4.578 | 0.014* | 0.050 |
| Aspartic acid | 14.775 | 0.000** | 0.010 | 8.173 | 0.001** | 0.010 | 10.146 | 0.000** | 0.010 |
| Asparagine | 8.983 | 0.004** | 0.010 | 6.092 | 0.004** | 0.010 | 28.255 | 0.000** | 0.010 |
| Cysteine | 15.492 | 0.000** | 0.010 | 0.168 | 0.846^ns^ | 0.050 | 0.379 | 0.686^ns^ | 0.050 |
| Glutamic acid | 2.951 | 0.090^ns^ | 0.050 | 3.643 | 0.031* | 0.050 | 4.022 | 0.022* | 0.050 |
| Glutamine | 1.323 | 0.254^ns^ | 0.050 | 2.362 | 0.102^ns^ | 0.050 | 0.565 | 0.571^ns^ | 0.050 |
| Glycine | 0.676 | 0.414^ns^ | 0.050 | 4.813 | 0.011* | 0.050 | 3.982 | 0.023* | 0.050 |
| Proline | 12.564 | 0.001** | 0.010 | 4.680 | 0.012* | 0.050 | 4.767 | 0.012* | 0.050 |
| Serine | 0.789 | 0.378^ns^ | 0.050 | 4.216 | 0.019* | 0.050 | 4.339 | 0.017* | 0.050 |
| Tyrosine | 0.013 | 0.911^ns^ | 0.050 | 10.712 | 0.000** | 0.010 | 5.695 | 0.005** | 0.010 |
| Histidine | 0.366 | 0.547^ns^ | 0.050 | 4.316 | 0.017* | 0.050 | 4.278 | 0.018* | 0.050 |
| Lysine | 7.384 | 0.008** | 0.010 | 4.120 | 0.020* | 0.050 | 1.533 | 0.223^ns^ | 0.050 |
| Threonine | 0.310 | 0.579^ns^ | 0.050 | 7.157 | 0.002** | 0.010 | 0.506 | 0.605^ns^ | 0.050 |
| Methionine | 1.155 | 0.286^ns^ | 0.050 | 4.814 | 0.011* | 0.050 | 1.770 | 0.178^ns^ | 0.050 |
| Phenylalanine | 5.941 | 0.017* | 0.050 | 3.011 | 0.056^ns^ | 0.050 | 0.005 | 0.995^ns^ | 0.050 |
| Leucine | 33.894 | 0.000** | 0.010 | 4.934 | 0.010* | 0.050 | 0.031 | 0.970^ns^ | 0.050 |
| Isoleucine | 5.172 | 0.026* | 0.050 | 2.086 | 0.132^ns^ | 0.050 | 11.570 | 0.000** | 0.010 |
| Valine | 12.089 | 0.001** | 0.010 | 4.707 | 0.012* | 0.050 | 0.374 | 0.689^ns^ | 0.050 |

Two-way ANOVA results for diet type (normal diet vs. high-protein diet), probiotic dose (0, 1 × 10^7^, 1 × 10^9^ CFU/day), and their interaction. Significance codes: ns = not significant; * = *p* < 0.05; ** = *p* < 0.01.

**Supplementary Table S8.** Comparison of the area under the curve (AUC) [nmol/mL • 240 min] of postprandial blood amino acids in rats.

| Amino acids | Control | AUC_ST4 |
| --- | --- | --- |
| Alanine | 3419.66 ± 272.93 | 4579.01 ± 583.89 |
| Arginine | 393.43 ± 33.34 | 300.08 ± 14.34 |
| Aspartic acid | 52.29 ± 6.27 | 52.29 ± 11.61 |
| Asparagine | 459.34 ± 29.36 | 451.64 ± 27.02 |
| Cysteine | 136.09 ± 6.26 | 283.06 ± 5.82^**^ |
| Glutamic acid | 965.50 ± 61.97 | 927.40 ± 92.20 |
| Glutamine | 2121.97 ± 159.64 | 3319.62 ± 99.30^*^ |
| Glycine | 2314.92 ± 170.60 | 2359.74 ± 86.83 |
| Proline | 718.88 ± 47.09 | 2266.86 ± 220.89^*^ |
| Serine | 622.14 ± 54.88 | 682.87 ± 41.28 |
| Tyrosine | 177.42 ± 16.14 | 304.67 ± 29.08^*^ |
| Histidine^†^ | 111.47 ± 1.40 | 144.72 ± 15.66 |
| Lysine^†^ | 1466.06 ± 11.17 | 3335.50 ± 237.55^**^ |
| Threonine^†^ | 849.76 ± 48.46 | 1122.01 ± 119.57 |
| Methionine^†^ | 248.92 ± 17.01 | 521.13 ± 14.75^**^ |
| Phenylalanine^†^ | 281.00 ± 6.45 | 355.61 ± 11.94^*^ |
| Leucine^†^ | 628.86 ± 35.30 | 916.88 ± 88.18 |
| Isoleucine^†^ | 429.19 ± 65.17 | 535.41 ± 57.92 |
| Valine^†^ | 1253.78 ± 85.49 | 1495.05 ± 136.19 |

Each value is presented as mean ± standard deviation (SD).

^*^ Significant at *p* < 0.05; ^**^significant at *p* < 0.01 compared with control group.

^†^ Indicates an amino acid categorized as essential.

**References**

Henderson, J. W., Ricker, R. D., Bidlingermeyer, B. A., and Woodward, C. (2000). Rapid, accurate, sensitive, and reproducible HPLC analysis of amino acids. https://www.agilent.com/cs/library/chromatograms/59801193.pdf [Accessed August 5, 2024].

LabDiet®. (2022). Laboratory Rodent Diet 5001. https://www.labdiet.com/getmedia/edc7a620-0240-4a7a-98fa-b3d69833f9ea/5001.pdf?ext=.pdf [Accessed August 5, 2024].

Weisburg, W. G., Barns, S. M., Pelletier, D. A., and Lane, D. J. (1991). 16S ribosomal DNA amplification for phylogenetic study. *J. Bacteriol.* 173:697-703. doi: 10.1128/jb.173.2.697-703.1991
